# Supplementary material for: Splice-Junction-Based Mapping of Alternative Isoforms in the Human Proteome
Source: Cell Rep. Author manuscript; Available in PMC 2020 Jan 15. (PMC6961840; doi:10.1016/j.celrep.2019.11.026)
Supplement: 2 [file NIHMS1546469-supplement-2.zip › DF1/PXD009737/TestisLysC/TestisLysC_22_RPS6KB1_KQAFPMISKRPEHLRMNAEK.pdf]

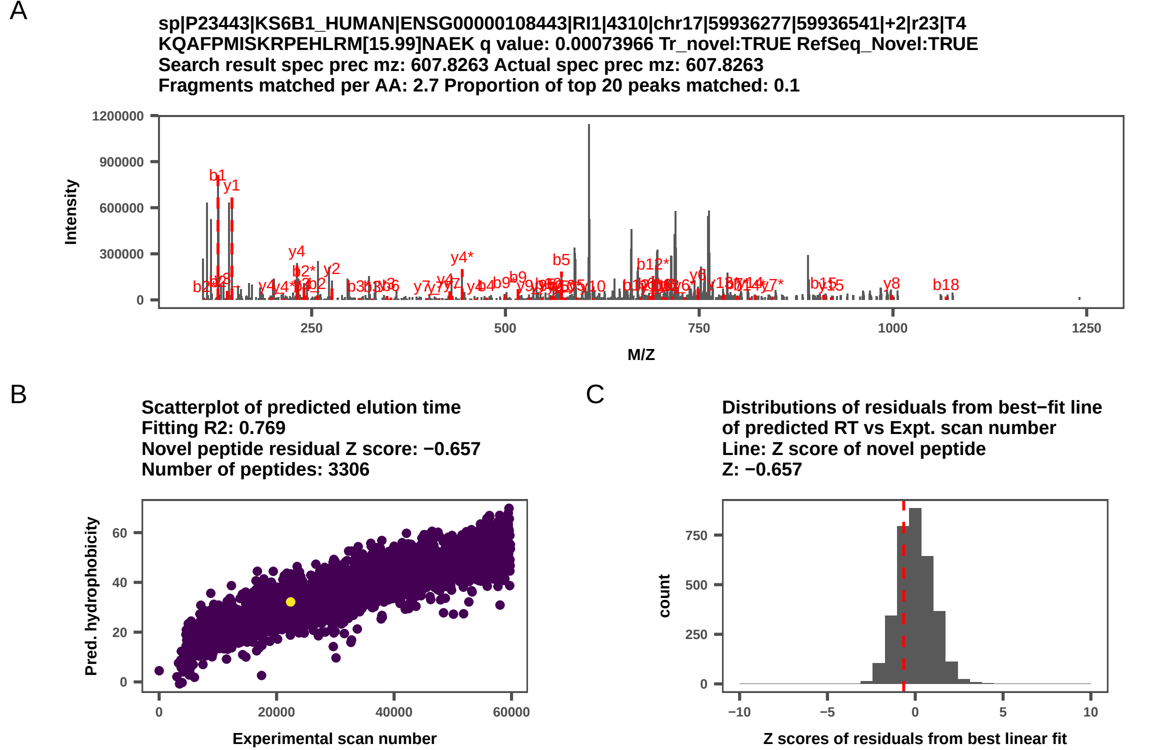
A
sp|P23443|KS6B1_HUMAN|ENSG00000108443|RI1|4310|chr17|59936277|59936541|+2|r23|T4
KQAFPMISKRPEHLRM[15.99]NAEK q value: 0.00073966 Tr_novel:TRUE RefSeq_Novel:TRUE
Search result spec prec mz: 607.8263 Actual spec prec mz: 607.8263
Fragments matched per AA: 2.7 Proportion of top 20 peaks matched: 0.1
Intensity
1200000
900000
600000
300000
0
b1
y1
y4
y4*
b5
b12*
y2
y6
b9
b15
y8
b18
250
500
750
1000
1250
M/Z
B
Scatterplot of predicted elution time
Fitting R2: 0.769
Novel peptide residual Z score: −0.657
Number of peptides: 3306
Pred. hydrophobicity
60
40
20
0
0
20000
40000
60000
Experimental scan number
C
Distributions of residuals from best−fit line
of predicted RT vs Expt. scan number
Line: Z score of novel peptide
Z: −0.657
count
750
500
250
0
−10
−5
0
5
10
Z scores of residuals from best linear fit
